# Supplementary material for: An Integrative Study of Aortic mRNA/miRNA Longitudinal Changes in Long-Term LVAD Support
Source: Int J Mol Sci. 2021 Jul 10;22(14):7414. doi: 10.3390/ijms22147414 (PMC8303892; doi:10.3390/ijms22147414)

Figure S1 Principal component analysis of expression profile. Samples before (IM) and after (EX) LVAD support.

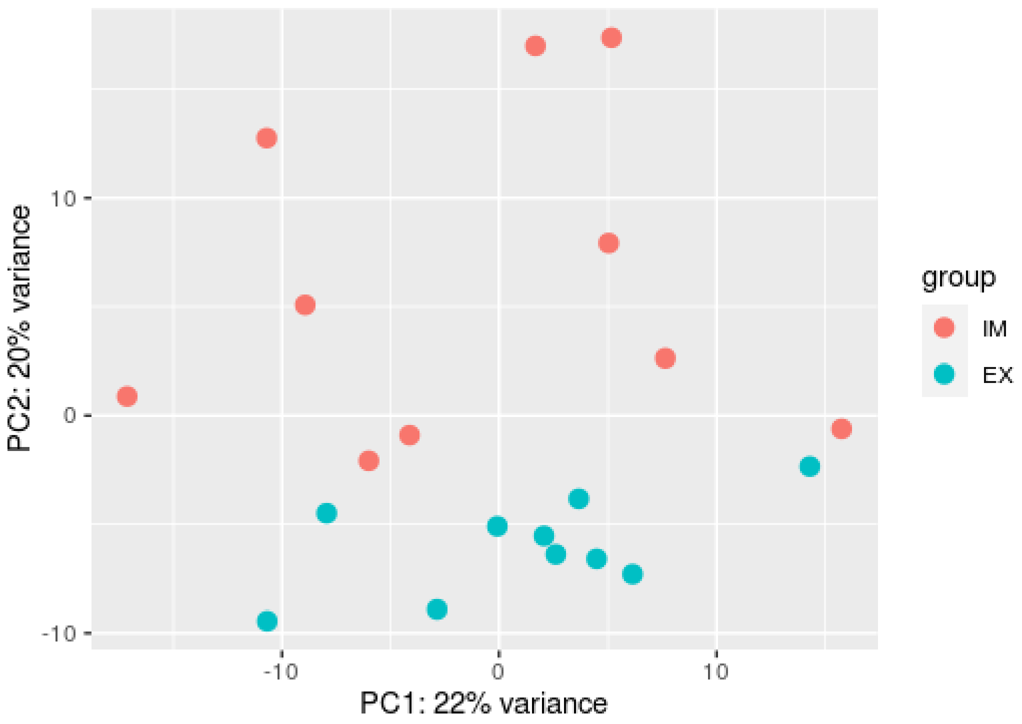

Figure S2 Heatmap and hierarchical structure of twenty overlapping mRNAs across 20 human aortic samples. IM depict before LVAD, EX depict after LVAD support

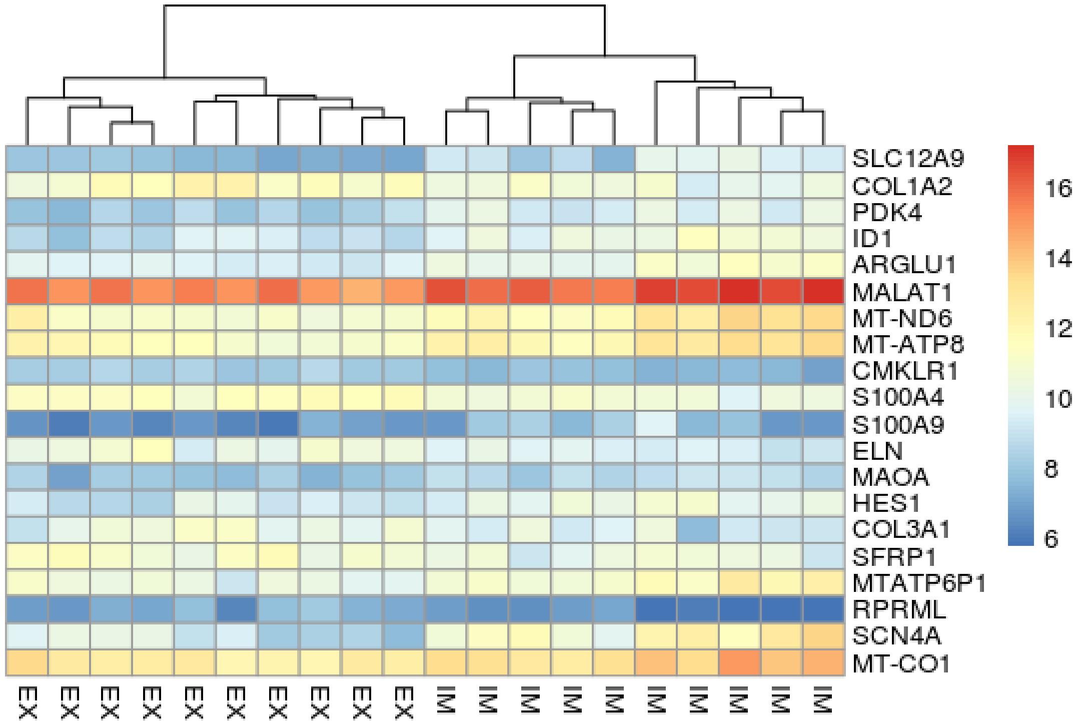

Figure S3 ***plotMA*** shows the log2 fold changes attributable to a given variable over the mean of normalized counts for all the samples in the *DESeqDataSet*. Red colored points demonstrate the adjusted *p* value is less than 0.1

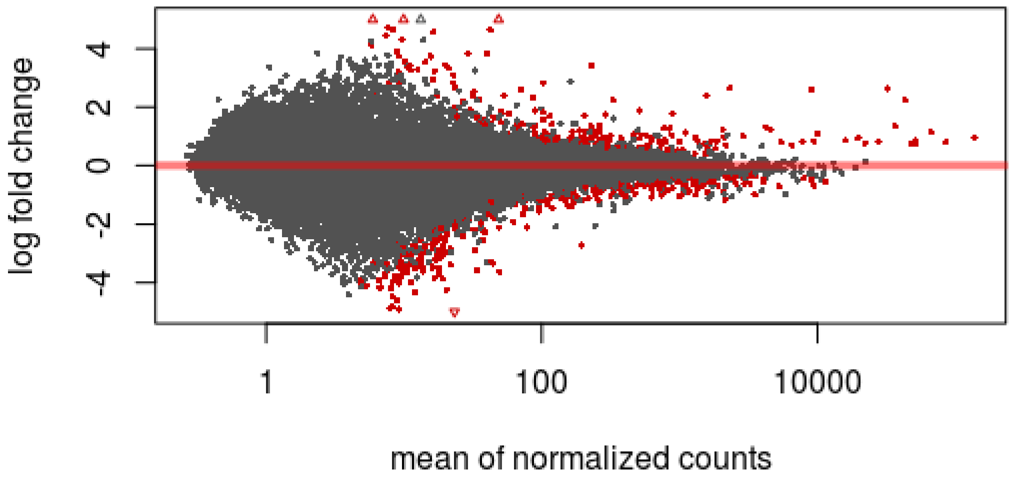

Supplement: Supplementary file 1 [file ijms-22-07414-s001.zip › Supplementary Figures.docx.pdf]
